# Effect of *Spirulina platensis* on growth, hematological, biochemical, and immunological parameters of Nile tilapia (*Oreochromis niloticus*)

**DOI:** 10.1007/s11250-023-03690-5

**Published:** 2023-07-27

**Authors:** Ibrahim M. I. Youssef, Elham S. E. Saleh, Samar S. Tawfeek, Asmaa A. A. Abdel-Fadeel, Abdel-Razik H. Abdel-Razik, Asmaa S. A. Abdel-Daim

**Affiliations:** 1 Department of Nutrition and Clinical Nutrition, Faculty of Veterinary Medicine, Beni-Suef University, Beni-Suef, 62511 Egypt; 2 Department of Histology, Faculty of Veterinary Medicine, Beni-Suef University, Beni-Suef, 62511 Egypt

**Keywords:** Nile tilapia, *Spirulina platensis*, Productive performance, Intestinal morphometry, Immune response

## Abstract

This study was conducted to evaluate the effects of *Spirulina platensis* in Nile tilapia diets on growth performance, blood hematological and biochemical parameters, immunological status, and intestinal histomorphometry. A total of 228 fish were randomly allocated into four groups with triplicates (19 fish per replicate). The first group was fed the control diet, which contained no Spirulina supplementation. The other three groups were fed diets containing graded levels of powdered Spirulina: 2.5%, 5.0%, and 10.0% in the second, third, and fourth group, respectively. *S. platensis* was added to the diets partially substituting the fish meal content. The experiment lasted for 8 weeks. The results showed that dietary Spirulina supplementation improved (*P* < 0.05) the body weight and length, weight gain, specific growth rate, condition factor, and feed conversion efficiency. Moreover, Spirulina increased significantly (*P* < 0.05) the hemoglobin, PCV, RBCs, and WBCs count. Also, it increased the lymphocytes, eosinophils, IgM level, lysozyme activity, and phagocytic activity in the blood. Additionally, the Spirulina raised (*P* < 0.05) the serum albumin level but reduced (*P* < 0.05) the creatinine and urea levels. The addition of Spirulina increased (*P* < 0.05) the height and width of intestinal villi and the lymphocytes and goblet cells count in the intestine. The obtained results were increased by increasing the inclusion level of Spirulina, especially for body weight and length, weight gain, FCR, phagocytic activity, and intestinal parameters. In conclusion, supplementing *S. platensis* can improve the growth performance of fish. Moreover, it can stimulate the immunity of fish through increasing the level of immunological blood indicators (IgM, lysozyme, phagocytic activity, lymphocytes, and eosinophils) as well as the local intestinal immunity (lymphocytes and goblet cells). So, it can be recommended to use *S. platensis* in fish diets not only to improve the growth performance but also to enhance the immune status.

## Introduction

Nowadays, the demand for aquaculture has been increased as one of the main sources of rational animal protein that required for human food (FAO, 2018; Mosha et al., 2020a). Generally, fish species shows relatively high protein demand in the diet. Fish meal and soybean meal are the main protein ingredients in fish diets. These protein sources are the most expensive feeds and are not sometimes available (Kristofersson and Anderson, 2006). Therefore, the need to search for alternative protein sources enhances the scientific community to find viable and accessible solutions (Sanz et al., 2000, Thum et al., 2022). Novel proteins are of major concern in the aquaculture feed industry. Due to the continuous increase in the cost of fish meal, many studies have started the evaluation of the economic feasibility and optimum use of these novel proteins as fish meal substitutes (Soler-Vila et al., 2009; Mosha, 2019; Zhang et al., 2020). Macro- and microalgae have been used as dietary supplements to improve the nutritional performance and health status of farmed fish species (Güroy et al., 2011). *S. platensis* is a fast-growing cyanobacterium of large size (0.5 mm) and is considered as a possible alternative protein source for cultured fish (Abdel-Latif et al., 2022). This is due to it is regarded as a rich source of protein, essential amino acids, vitamins, minerals, essential fatty acids contents (gamma-linolenic acid), antioxidant pigments such as carotenoids (C-phycocyanin), antimicrobial properties, and anticancer activity (Zhang et al., 2020; Wan et al., 2021). Spirulina has been produced commercially for about 20 years and is sold mostly as a human food additive, medicine, and food coloring agent. Nonetheless, about 30% of the current world algal output is sold for animal feeding applications, and over 50% of the current global production of Spirulina is used as a feed supplement (Rando and Rene, 2020). Many studies showed a significant improvement of Spirulina in the growth rates, immunity, and diseases resistance of Nile tilapia (Abdel-Tawwab and Ahmad, 2009; Amer, 2016; Mahmoud et al., 2018; Mosha et al., 2020b). In addition, the use of Spirulina as an immune stimulant and antioxidant can reduce antibiotics usage and stress conditions in fish (Wu et al., 2016; Adel et al., 2016).

Consequently, Spirulina is protruding as a cost-effective mean of enhancing animal productivity for a sustainable and feasible food safety future. The present study was conducted on tilapia fish to test partial replacement of fish meal by *S. platensis*. The effects of this beneficial cyanobacterium on growth performance and hematological, biochemical, and immunological parameters of the blood as well as the histomorphometry and immunity of the intestine were also studied.

## Materials and methods

### Experimental design and fish feeding

A total of 228 fish with starting body weights of about 20.0 ± 0.35 g were randomly distributed into four groups. Each group was divided into 3 replicates, and each replicate contained 19 fish. The first group was fed the control diet which did not contain *S. platensis* supplementation. The other three groups were fed diets containing graded levels of Spirulina powder: 2.5%, 5.0%, and 10.0% in the second, third, and fourth group, respectively. The control and experimental diets were identical in protein (30% CP) and energy (3060 kcal/kg DE). The DE of fish feeds was calculated as described by previous studies (Smith, 1971; Page and Andrews, 1973). The diets were formulated to satisfy the nutrient requirements of fish according to the guidelines of NRC (2011). The experiment lasted for 8 weeks. *S. platensis* was added to the diets partially replacing the fish meal content, and it was obtained from Biotech International Research and Development (BIRD) Centre, Mansoura, Egypt. Fish meal was reduced by about 2.50, 4.50, and 7.50% for 2.50, 5.0, and 10.0% Spirulina-supplemented diets, respectively, with slight adjusting the other ingredients to satisfy the nutrient requirements of tilapia. During the experimental period, the tilapia fish were fed 3 times daily (9:00, 12:00, and 15:00 h) with a feeding dose of 4% and 3% of body weight for the first 2 weeks and the last 6 weeks, respectively. Spirulina was analyzed for the nutrients content, and it was found to contain 51.0% CP, 8.80% CF, 6.75% ether extract, 11.70% ash, 2.17% methionine, 3.0% lysine, 0.28% calcium, and 0.38% phosphorus. Moreover, the ingredients used in formulating the diets were analyzed for the proximate chemical composition according to AOAC (2005; Table 1), and consequently, the diets were formulated. The ingredients’ composition and chemical analyses of the different diets are shown in Table 2. Before the beginning of experiment, the tilapia were fed the control diet and adapted to laboratory conditions for two weeks. Fish were stocked in 12 glass aquaria with dimensions of 82 cm length × 40 cm width × 44 cm depth for each aquarium. Every aquarium was filled with 95 l of dechlorinated tap water. The aquaria were partially cleaned by siphoning the accumulated excreta daily, and about half of the water content was changed once or twice weekly according to the water state. Water temperature and pH in aquaria were determined daily by using a thermometer and pH meter, and they were in the range of 27 to 28 °C and 7.28 to 7.76, respectively, throughout the experiment. Air pumps were fitted to the aquaria throughout the experimental period for the aeration of the water. The mortality rates of fish were recorded daily throughout the experimental trial.

**Table 1:** Chemical composition (%) and energy value (kcal/kg) of feed ingredients on as-fed basis

Ingredient	Dry matter	Digestible energy^a^	Crude protein	Methionine	Lysine	Ether extract	Crude fiber	Ash	Nitrogen free extract^b^	Calcium	Total phosphorus	Available phosphorus^e^
Yellow corn, ground	87.53	2644.37	7.88	0.18	0.24	3.70	2.50	0.99	72.46	0.03	0.28	0.08
Soybean meal (44% CP)	90.37	3073.29	43.84	0.61	2.83	2.97	6.70	5.91	30.95	0.26	0.64	0.27
Fish meal (65% CP)	90.24	3819.66	65.45	1.77	4.81	8.60	0.95	11.39	3.85	5.19	2.88	1.44
Corn gluten meal (60% CP)	90.48	3556.92	60.38	1.90	1.07	1.75	1.45	1.49	25.41	0.07	0.44	0.14
Sunflower oil	99.90	8750.00	-	-	-	99.90	-	-	-	-	-	-
Carboxymethyl cellulose^c^	96.00	-	-	-	-	-	92.60	-	3.40	-	-	-
*Spirulina platensis* algae^d^	90.67	3235.15	51.00	2.17	3.00	6.75	8.80	11.70	12.42	0.28	0.38	0.38

^a^Digestible energy was estimated according to the following equation: digestible energy (kj/g) = 18.8 × CP + 37.7 × EE + 11.3 × NFE (Smith, 1971; Page and Andrews, 1973) for all ingredients except the DE of the vegetable oil obtained from NRC of fish (2011)

^b^Nitrogen-free extract was estimated using the following formula: nitrogen free extract = dry matter – (crude protein + crude fiber + ash + ether extract)

^c^Carboxymethyl cellulose is a purified ingredient

^d^Spirulina algae were analyzed for CP, CF, ether extract, and ash, whereas the other nutrients were obtained from the produced center and research studies (Tolba, 2014 and Zhang et al., 2020)

^e^Available phosphorus values were obtained from NRC of fish (2011)

**Table 2:** Physical and chemical composition of the experimental diets (as-fed)

Composition	Group			
	Spirulina levels in the diet (%)			
	0	2.5	5.0	10.0
Physical composition, %				
Yellow corn	41.90	40.44	39.03	35.94
Soybean meal (44% CP)	39.00	39.00	39.00	41.00
Fish meal (65% CP)	10.50	8.00	6.00	3.00
Corn gluten meal (60% CP)	4.52	5.30	5.54	3.53
Sunflower oil	2.25	2.54	2.82	3.33
Monocalcium phosphate	0.32	0.45	0.55	0.66
Limestone, ground	0.10	0.32	0.54	0.91
Common salt	0.20	0.21	0.25	0.27
Vitamin and mineral premix^a^	0.50	0.50	0.50	0.50
Carboxy methyl cellulose	0.50	0.50	0.50	0.50
Choline chloride	0.10	0.10	0.10	0.10
DL-methionine	0.06	0.09	0.12	0.21
Vitamin C	0.05	0.05	0.05	0.05
Spirulina platensis	-	2.50	5.00	10.00
Chemical composition (calculated)				
Digestible energy, kcal/ kg	3065.30	3065.20	3065.40	3065.50
Crude protein, %	30.00	30.00	30.00	30.00
Crude fiber, %	3.96	4.14	4.32	4.78
Ether extract, %	5.92	6.12	6.34	6.84
Methionine, %	0.64	0.64	0.64	0.64
Lysine, %	1.76	1.72	1.70	1.73
Calcium, %	0.66	0.64	0.64	0.64
Total Phosphorus, %	0.62	0.58	0.55	0.49
Available phosphorus, %	0.36	0.36	0.36	0.36
Sodium, %	0.14	0.14	0.14	0.14

^a^Vitamin and mineral premix is composed of: vitamin A acetate (6,250,000 I.U./kg), vitamin D3 (cholecalciferol) (2,500,000 I.U./kg), vitamin E (α-tocoopherol) (25,000 mg/kg), vitamin K3 (menadione sodium bisulfite) (1750 mg/kg), vitamin B1(500 mg/kg), vitamin B2 (2750 mg/kg), vitamin B6 (1250 mg/kg), vitamin B12 (10 mg/kg), nicotinic acid (niacin) (20,000 mg/kg), calcium pantothenate (5000 mg/kg), folic acid (500 mg/kg), biotin (50 mg/kg), iron sulfate (22,000 mg/kg), manganese oxide (31,000 mg/ kg), copper sulfate (2500 mg/kg), zinc oxide (37,500 mg/kg), potassium iodide (650 mg/kg), selenium selenite (113 mg/kg), cobaltous sulfate (50 mg/kg), ethoxyquin (250 mg/kg), wheat bran (carrier) (120 gm/kg), and limestone (carrier) (up to 1 kg)

### Growth performance

The feed intake (FI) of fish was measured daily, whereas the weight and length of fish were recorded weekly throughout the experiment. The fish were dried using a clean filter paper before weighing and then weighed by using a digital balance. The body weight gain (BWG) was determined by taking the difference between the initial and final weight. The body length of tilapia was monitored during its weighing, using a measuring board as represented by Lagler (1978). The length was determined as the distance from the muzzle to the starting of the caudal fin. The weight and length of fish were recorded to the nearest 0.1g and mm, respectively. The condition factor (*K*) was considered as *K* = 100 × *W*/*L*^3^, where *W* is the total body weight (g) and *L* is the total fish length (cm). The specific growth rate of tilapia (SGR, % body weight/day) was assessed as SGR = 100 [(ln *W*_t_ – ln *W*_0_) /*t*], where *W*_0_ and *W*_t_ are the starting and final weights of live body (g), respectively, ln is the natural logarithm, and (*t*) is the feeding interval in days. Feed conversion ratio (FCR) was estimated as FI (g)/BWG (g). The protein efficiency ratio (PER) of fish is realized as the weight gain for every unit weight of dietary protein consumed, and it was determined by dividing the weight gain (g) by the protein intake (g).

### Hematological, biochemical, and immunological blood analyses

At the end of the experimental period, blood samples were gathered, through the tail blood vessels, from 6 tilapia per treatment (two fish/replicate) using a sterile syringe. Every sample was separated into two fractions: The first fraction was transported into a 2-ml sterile test tube with heparin for hematological examination, and the second one remained in a 2-ml plain Eppendorf tube for separation of the serum. Blood was kept to clot at 4 °C for 1 h. Afterward, the tubes were centrifuged at 1811*g* for 15 min, for serum separation. Serum was taken in Eppendorf tubes and stored at – 20 °C until analyzed chemically. The following hematological parameters were measured: red blood cells (RBCs), hemoglobin, packed cell volume (PCV), and total and differential leucocytic counts (neutrophils, lymphocytes, monocytes, and eosinophils) using an automatic blood cell counter. The differential leucocytes count was measured to evaluate the effect of algae on the immune cells’ status in the blood.

At the time of analysis, the serum samples were dissolved and colorimetrically analyzed for total protein, albumin, glutamic oxaloacetic transaminase (GOT), glutamic pyruvic transaminase (GPT), glucose, total cholesterol, triglycerides, creatinine, and urea using commercial test kits (Diamond diagnostic company, Egypt). The globulin was determined by subtracting albumin amounts from total protein. Albumin/globulin (A/G) ratio was estimated by dividing albumin values by globulin amounts.

Regarding to the immunological blood parameters, the immune globulin M (IgM) and lysozyme activity were measured in the serum by using ELISA (enzyme-linked immunosorbent assay) technique according to Amer (2016) and Lawton (2006). In addition, the phagocytic activity was determined using *E. coli* as foreign bodies, as described by Bedasso (2017).

### Intestinal histomorphometry and immunity

At the end of the trial, 6 tilapia fish were randomly selected from each treatment (two fish/replicate) for intestinal histomorphometry and immunity determination. After deep anesthesia using 40% ethyl alcohol, the abdomen was expounded, and samples from proximal and distal portions of the intestine were collected. The samples were fixed in a 10% neutral buffered formalin. Afterward, the samples were dehydrated by ascending concentrations of ethanol, and then cleared in xylene, impregnated in soft paraffin, and then implanted in hard paraffin for blocking. Serial histological slices of 4–6 μm width were cut by a microtome and mounted on clean and dry glass slides. The obtained sections were stained with Harris Hematoxylin and Eosin. The sections were taken serially from each intestinal part. The diameter and length of ten intestinal villi, as well as the depth of ten crypts, were determined from each section per segment. The mean was calculated from these values. All histomorphometry investigations were carried out according to Sikandar et al. (2017). The measurements were achieved with the aid of the Image J analysis software program, Microsoft Company, using LEICA (DFC290 HD system digital camera, Heerbrugg, Switzerland) linked to the light microscope using 10 and 1000 × objective lens. In addition to previous measurements, the numbers of goblet cells and lymphocytes in the intestinal epithelium were determined according to Bancroft and Gamble (2008).

### Statistical analysis

The statistical analysis of the results was performed using SAS statistical program (SAS Institute, 2002). The data were assessed using the general linear models (GLM) procedure for analysis of variance. The results were analyzed using ANOVA tests followed by Duncan’s multiple range test to determine the differences among the treatments. The growth performance results were subjected to two-way ANOVA with repeated measurements throughout different weeks of the experiment, while other results were analyzed by one-way ANOVA test after the end of the feeding trial. Differences were considered to be significant when p value was < 0.05. The results are presented as means with standard deviation (means ±SD).

## Results

### Growth performance

The results of fish growth performance are shown in Tables 3 and 4. The data of biweekly measurements only as well as that of total experimental period are presented in these tables. The body weight and weight gain of fish were increased (*P* < 0.05) with increasing the level of *S. platensis* in the diets. The SGR was higher (*P* < 0.05) in Spirulina-supplemented groups when compared to the control. It was found that the SGR was the highest in 5.0 and 10.0% Spirulina, followed by 2.50% treatment. The length of fish was significantly (*P* < 0.05) higher in 10.0% Spirulina, but numerically higher in 2.50 and 5.0% levels than the control. The condition factor was higher (*P* < 0.05) in all Spirulina-supplemented treatments than the control group. However, there were no significant (*P* > 0.05) differences in the feed intake among the experimental groups. Nevertheless, the feed intake was found to be numerically increased by increasing the Spirulina level in the diets. Moreover, the FCR was lower in Spirulina-supplemented treatments than the control. The FCR was improved by 5.0 and 10.0% Spirulina groups, followed by 2.50% group. The PER was higher significantly (*P* < 0.05) in 5.0 and 10.0% Spirulina and numerically in 2.50% treatment when compared to the control. However, there was no difference (*P* > 0.05) in the mortality rate among the treatments.

**Table 3:** Body weight and length as well as the condition factor of Nile tilapia fed diets containing graded levels of *Spirulina platensis* throughout the experimental period

Parameters	Spirulina levels in the diet (%)	Duration of experiment (week)					*P* value		
		0	2	4	6	8	Treat.	Time	Tr. × T
Body weight (g)	0	21.71 ± 0.35^aD^	26.85 ± 0.75^aD^	31.75 ± 0.58^aC^	38.23 ± 0.49^bB^	46.02 ± 0.66^dA^	< 0.001	< 0.001	< 0.001
	2.5	21.70 ± 0.83^aD^	26.73 ± 1.68^aD^	32.32 ± 1.77^aC^	39.38 ± 0.86^bB^	48.21 ± 0.28^cA^			
	5.0	21.89 ± 0.27^aD^	26.15 ± 1.92^aD^	32.46 ± 0.64^aC^	40.11 ± 0.82^abB^	50.71 ± 0.07^bA^			
	10.0	22.32 ± 0.39^a^	27.18 ± 0.72^a^	33.34 ± 1.03^a^	41.23 ± 0.86^a^	53.22 ± 0.26^a^			
Body length (cm)	0	11.00 ± 0.08^aB^	11.71 ± 0.11^aB^	12.41 ± 0.10^aA^	13.20 ± 0.13^aA^	13.85 ± 0.08^bA^	0.030	0.041	0.038
	2.5	10.98 ± 0.18^aB^	11.71 ± 0.25^aB^	12.44 ± 0.21^aA^	13.29 ± 0.09^aA^	13.87 ± 0.09^bA^			
	5.0	11.02 ± 0.08^aB^	11.62 ± 0.33^aB^	12.48 ± 0.11^aA^	13.34 ± 0.13^aA^	14.01 ± 0.06^abA^			
	10.0	11.06 ± 0.14^aB^	11.75 ± 0.14^aB^	12.55 ± 0.19^aA^	13.44 ± 0.18^aB^	14.16 ± 0.18^aA^			
Condition factor (g/cm^3^)	0	1.63 ± 0.04^aA^	1.66 ± 0.02^aA^	1.65 ± 0.01^aA^	1.66 ± 0.05^aA^	1.73 ± 0.03^bA^	0.005	0.036	0.043
	2.5	1.63 ± 0.03^aB^	1.65 ± 0.02^aB^	1.66 ± 0.02^aB^	1.67 ± 0.04^aB^	1.79 ± 0.03^aA^			
	5.0	1.63 ± 0.03^aB^	1.65 ± 0.04^aB^	1.66 ± 0.02^aB^	1.68 ± 0.08^aAB^	1.82 ± 0.00^aA^			
	10.0	1.63 ± 0.04^aB^	1.66 ± 0.03^aB^	1.67 ± 0.02^aB^	1.69 ± 0.07^aB^	1.83 ± 0.03^aA^			

^a,b^Means within the same column (within each parameter) with different superscripts are significantly different (*P* < 0.05)

^A,B^Means within the same row with different superscripts are significantly different (*P* < 0.05)

**Table 4:** Growth performance and feed utilization of Nile tilapia fed diets containing graded levels of *Spirulina platensis* throughout the experimental period (biweekly and total values)

Parameters	Spirulina levels in the diet (%)	Duration of experiment (week)						*P* value		
		0	2	4	6	8	total (0-8)	Treat.	Time	Tr. x T
Body weight gain (g)	0	2.74 ± 0.16^aB^	2.40 ± 0.24^aB^	2.72 ± 0.03^aB^	3.37 ± 0.12^bA^	3.98 ± 0.58^bA^	24.31 ± 0.58^d^	< 0.001	< 0.001	< 0.001
	2.5	2.48 ± 0.59^aC^	2.56 ± 1.07^aC^	3.28 ± 0.75^aB^	3.63 ± 0.06^abB^	4.37 ± 0.72^bA^	26.51 ± 0.89^c^			
	5.0	1.95 ± 0.74^aC^	2.31 ± 0.91^aC^	3.45 ± 0.24^aB^	4.09 ± 0.44^aA^	4.92 ± 0.43^bA^	28.81 ± 0.25^b^			
	10.0	2.78 ± 0.63^aD^	2.08 ± 0.49^aD^	3.53 ± 0.40^aC^	4.09 ± 0.26^aB^	6.07 ± 0.30^aA^	30.90 ± 0.59^a^			
SGR (% BW/ day)	0	1.98 ± 0.08^aA^	1.56 ± 0.12^aA^	1.49 ± 0.01^aA^	1.54 ± 0.06^bA^	1.51 ± 0.22^cA^	1.56 ± 0.03^c^	0.002	0.231	0.142
	2.5	1.80 ± 0.43^aA^	1.66 ± 0.65^aA^	1.79 ± 0.44^aA^	1.61 ± 0.03^abA^	1.59 ± 0.28^bA^	1.66 ± 0.08^b^			
	5.0	1.41 ± 0.50^aA^	1.52 ± 0.51^aA^	1.88 ± 0.16^aA^	1.79 ± 0.16^aA^	1.70 ± 0.16^abA^	1.75 ± 0.02^ab^			
	10.0	1.95 ± 0.41^aA^	1.33 ± 0.34^aA^	1.87 ± 0.20^aA^	1.74 ± 0.10^aA^	2.02 ± 0.11^aA^	1.81 ± 0.04^a^			
Feed intake (g)	0	3.54 ± 0.08^aB^	3.63 ± 0.14^aB^	5.22 ± 0.10^aA^	6.27 ± 0.10^bA^	7.57 ± 0.10^cA^	43.04 ± 0.26^a^	0.251	0.012	0.306
	2.5	3.52 ± 0.36^aB^	3.63 ± 0.58^aB^	5.53 ± 0.42^aA^	6.44 ± 0.15^bA^	7.73 ± 0.13^cA^	43.83 ± 1.92^a^			
	5.0	3.01 ± 0.01^aB^	3.61 ± 0.91^aB^	5.35 ± 0.36^aA^	6.48 ± 0.07^bA^	8.24 ± 0.08^bA^	43.91 ± 1.90^a^			
	10.0	3.58 ± 0.12^aC^	3.64 ± 0.26^aC^	5.46 ± 0.23^aB^	6.69 ± 0.13^aA^	8.49 ± 0.07^aA^	45.68 ± 1.26^a^			
FCR	0	1.30 ± 0.11^aB^	1.53 ± 0.22^aB^	1.92 ± 0.02^aA^	1.86 ± 0.08^aA^	1.93 ± 0.82^aA^	1.77 ± 0.05^a^	0.002	0.034	0.010
	2.5	1.47 ± 0.33^aB^	1.52 ± 0.36^aB^	1.71 ± 0.56^aA^	1.77 ± 0.03^abA^	1.80 ± 0.28^abA^	1.65 ± 0.07^b^			
	5.0	1.69 ± 0.59^aA^	1.63 ± 0.29^aA^	1.56 ± 0.20^aA^	1.59 ± 0.15^bA^	1.68 ± 0.16^abA^	1.52 ± 0.08^c^			
	10.0	1.33 ± 0.29^aB^	1.79 ± 0.28^aA^	1.55 ± 0.12^aAB^	1.64 ± 0.09^bA^	1.40 ± 0.08^bB^	1.48 ± 0.06^c^			
PER	0	2.58 ± 0.20^aA^	2.21 ± 0.29^aA^	1.74 ± 0.02^aB^	1.79 ± 0.08^bB^	1.76 ± 0.26^bB^	1.88 ± 0.05^b^	0.003	0.154	0.210
	2.5	2.34 ± 0.46^aA^	2.29 ± 0.60^aA^	2.08 ± 0.59^aA^	1.88 ± 0.03^abA^	1.88 ± 0.29^bA^	2.02 ± 0.08^b^			
	5.0	2.16 ± 0.82^aA^	2.09 ± 0.35^aA^	2.16 ± 0.26^aA^	2.10 ± 0.20^aA^	1.99 ± 0.19^abA^	2.19 ± 0.11^a^			
	10.0	2.58 ± 0.53^aA^	1.89 ± 0.33^aA^	2.15 ± 0.17^aA^	2.04 ± 0.12^aA^	2.38 ± 0.13^aA^	2.26 ± 0.09^a^			
Mortality rate (%)	0	3.51 ± 3.04^aA^	0.00 ± 0.00^aB^	0.00 ± 0.00^aB^	0.00 ± 0.00^aB^	0.00 ± 0.00^aB^	7.02 ± 3.04^a^	0.531	0.312	0.450
	2.5	1.75 ± 3.04^aA^	0.00 ± 0.00^aB^	1.75 ± 3.04^aB^	0.00 ± 0.00^aB^	0.00 ± 0.00^aB^	10.53 ± 0.00^a^			
	5.0	0.00±0.00^aB^	3.51 ± 6.08^aA^	1.96 ± 3.39^aA^	0.00 ± 0.00^aB^	0.00 ± 0.00^aB^	7.02 ± 8.04^a^			
	10.0	1.75 ± 3.04^aA^	0.00 ± 0.00^aA^	1.75 ± 3.04^aA^	0.00 ± 0.00^aA^	1.75 ± 3.04^aA^	5.26 ± 0.00^a^			

*SGR*, specific growth rate; *FCR*, feed conversion ratio; *PER*, protein efficiency ratio

^a,b^Means within the same column (within each parameter) with different superscripts are significantly different (*P* < 0.05)

^A,B^Means within the same row with different superscripts are significantly different (*P* < 0.05)

### Hematological, biochemical, and immunological blood analyses

The results of hematological and serum biochemical parameters are presented in Table 5. It was observed that the hemoglobin, PCV, RBCs, and WBCs count were significantly (*P*> 0.05) higher in the Spirulina-supplemented treatments than the control. However, there were no significant (*P* > 0.05) differences in the total protein, globulin, albumin/globulin ratio, glucose, triglycerides, cholesterol, GOT, and GPT among the experimental groups. Nevertheless, the serum albumin level was higher significantly (*P*> 0.05) in the 10.0% Spirulina treatment and numerically in 2.50 and 5.0% Spirulina when compared to the control. Moreover, the serum urea and creatinine levels were reduced (*P* < 0.05) by Spirulina supplementation.

**Table 5:** Hematological and serum biochemical parameters of Nile tilapia fed diets containing graded levels of *Spirulina platensis*

Parameters	Group				*P* value
	Spirulina levels in the diet (%)				
	0	2.5	5.0	10.0	
Hb (g/dl)	11.86± 0.84^b^	12.97± 0.12^a^	12.96± 0.08^a^	13.64 ± 0.63^a^	0.021
PCV (%)	33.10± 2.35^c^	43.20± 1.93^ab^	40.00± 1.60^b^	44.57± 1.70^a^	0.0004
RBCs (x10^6^/ μl)	2.10± 0.30^c^	3.33± 0.25^ab^	3.05± 0.15^b^	3.57± 0.21^a^	0.0003
WBCs (x10^3^/ μl)	45.01± 0.40^c^	59.70± 0.31^ab^	56.51± 0.15^b^	63.70± 0.45^a^	0.001
Total protein (g/dl)	5.72± 0.80^a^	6.12± 1.42^a^	5.58± 0.32^a^	5.82± 0.41^a^	0.884
Albumin (g/dl)	2.99± 0.01^b^	3.19± 0.28^ab^	3.22± 0.27^ab^	3.41± 0.17^a^	0.043
Globulin (g/dl)	2.73± 0.80^a^	2.93± 1.28^a^	2.36± 0.59^a^	2.41± 0.25^a^	0.809
A/G ratio	1.16± 0.30^a^	1.26± 0.61^a^	1.46± 0.56^a^	1.42± 0.08^a^	0.820
Glucose (mg/dl)	92.11± 9.74^a^	96.62± 4.85^a^	97.99± 10.87^a^	90.10± 11.61^a^	0.727
Triglycerides (mg/dl)	85.20± 7.41^a^	89.69± 3.41^a^	85.34± 16.04^a^	85.40± 8.51^a^	0.930
Cholesterol (mg/dl)	93.92± 7.08^a^	98.96± 3.02^a^	94.44± 16.39^a^	94.80± 8.57^a^	0.920
GOT (U/L)	28.76± 1.69^a^	28.30± 5.32^a^	24.46± 0.91^a^	24.91± 5.06^a^	0.425
GPT (U/L)	23.57± 1.39^a^	23.19± 4.37^a^	20.59± 0.74^a^	20.42± 4.15^a^	0.500
Creatinine (mg/dl)	0.69± 0.05^a^	0.51± 0.06^b^	0.40± 0.06^c^	0.39± 0.01^c^	0.0002
Urea (mg/dl)	23.63± 0.62^a^	19.86± 1.49^b^	17.03± 1.35^c^	16.55± 0.23^c^	0.0001

*Hb*, hemoglobin; *PCV*, packed cell volume; *RBCs*, red blood cells; *WBCs*, white blood cells; *A/G ratio*, albumin-to-globulin ratio; *GOT*, glutamic oxaloacetic transaminase; *GPT*, glutamic pyruvic transaminase

^a,b^Means within the same row with different superscripts are significantly different (*P* > 0.05)

The results of immunological blood parameters are shown in Table 6. Supplementation of Spirulina increased significantly (*P* < 0.05) lymphocytes count and IgM level. Moreover, there was a numerical increase in the eosinophils and lysozyme activity in the Spirulina-supplemented groups when compared to the control. However, the number of monocytes and neutrophils did not differ significantly (*P* > 0.05) among the experimental groups. The phagocytic activity was higher significantly (*P* < 0.05) in 10.0% Spirulina group and numerically in 2.50 and 5.0% Spirulina treatments.

**Table 6:** Differential leucocytic counts and immunological blood parameters of Nile tilapia fed diets containing graded levels of *Spirulina platensis*

Parameters	Group				*P* value
	Spirulina levels in the diet (%)				
	0	2.5	5.0	10.0	
Lymphocytes (%)	80.07 ± 2.61^b^	84.23 ± 1.66^a^	84.20 ± 2.07^a^	86.27 ± 3.40^a^	0.001
Monocytes (%)	2.67 ± 1.31^a^	2.15 ± 0.25^a^	2.47 ± 0.87^a^	2.20 ± 0.75^a^	0.876
Neutrophils (%)	12.60 ± 3.50^a^	13.03 ± 1.22^a^	12.83 ± 1.72^a^	11.70 ± 2.00^a^	0.893
Eosinophils (%)	0.83 ± 0.15^a^	0.87 ± 0.12^a^	0.85 ± 0.05^a^	0.85 ± 0.05^a^	0.983
IgM (μg/ml)	1.75 ± 0.03^b^	2.03 ± 0.41^a^	1.98 ± 0.01^a^	2.03 ± 0.17^a^	0.021
Lysozyme activity (ng/ml)	4.88 ± 0.36^a^	4.96 ± 0.36^a^	4.90 ± 0.02^a^	4.93 ± 0.16^a^	0.966
Phagocytic activity (%)	78.50 ± 2.50^b^	79.25 ± 0.25^b^	81.00 ± 2.00^b^	86.33 ± 1.53^a^	0.003

*IgM* immunoglobulin M

^a,b^Means within the same row with different superscripts are significantly different (*P* > 0.05)

### Intestinal histomorphometry and immunity

**Fig. 1:**
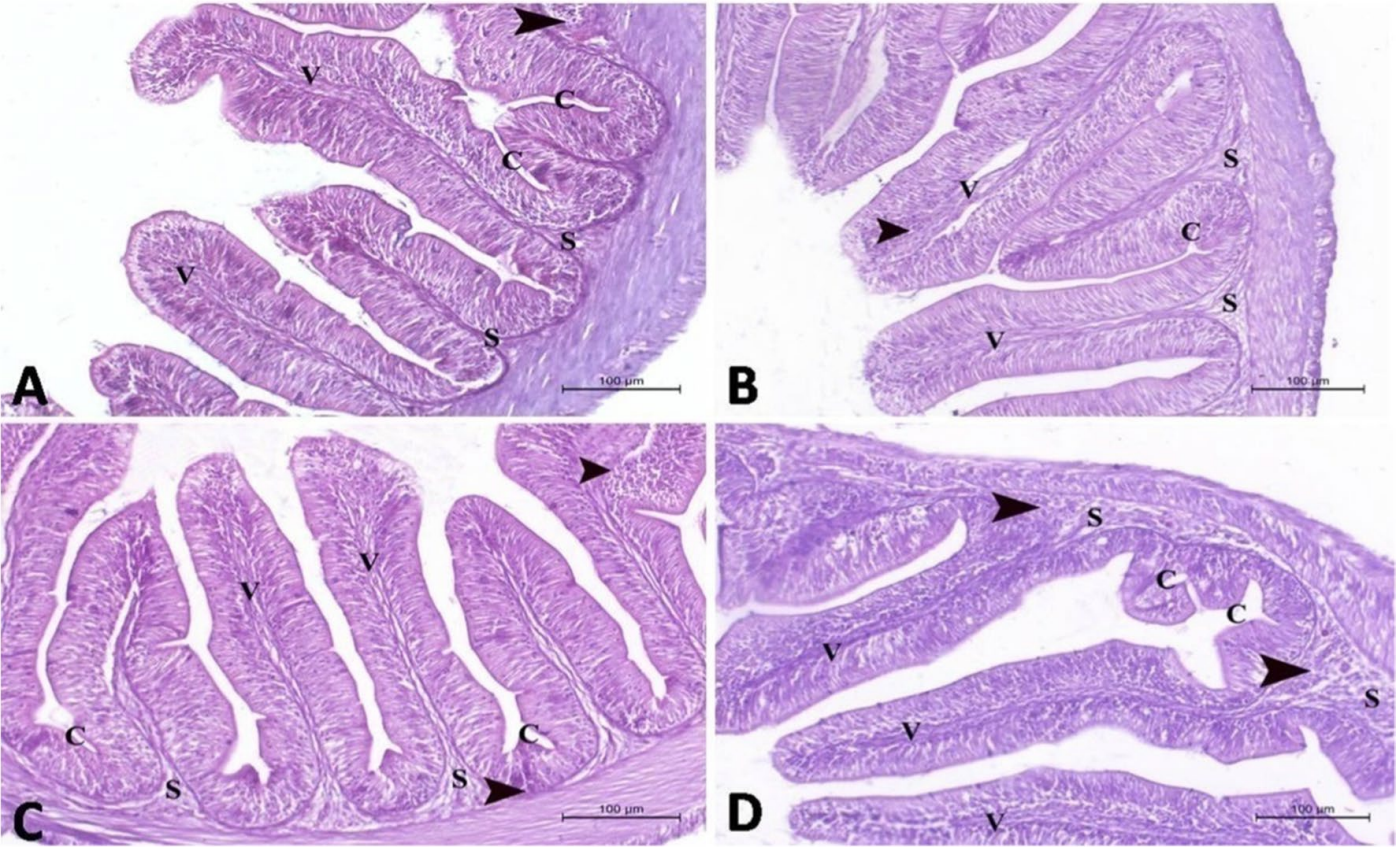
A photomicrograph of the proximal segment of intestine in Nile tilapia (H&E stain ×200) in **A** control group, **B** 2.5% Spirulina group, **C** 5.0% Spirulina group, and **D** 10.0% Spirulina group showing normal histological structure of the proximal segment of intestine with normal intestinal villi (V) of the intestinal mucosa which lined with columnar epithelium and incorporated goblet cells. The intestinal crypts (C) invaded in the submucosa (S) which contain aggregation of lymphocytes (arrow head). Note: the intestinal villi were increased in the length and width in **B**, **C**, and **D** groups, respectively, when compared to the control group. On the same way, the lymphocytes aggregations were increased in density in **B**, **C**, and **D** groups, respectively, when compared to the control

**Fig. 2:**
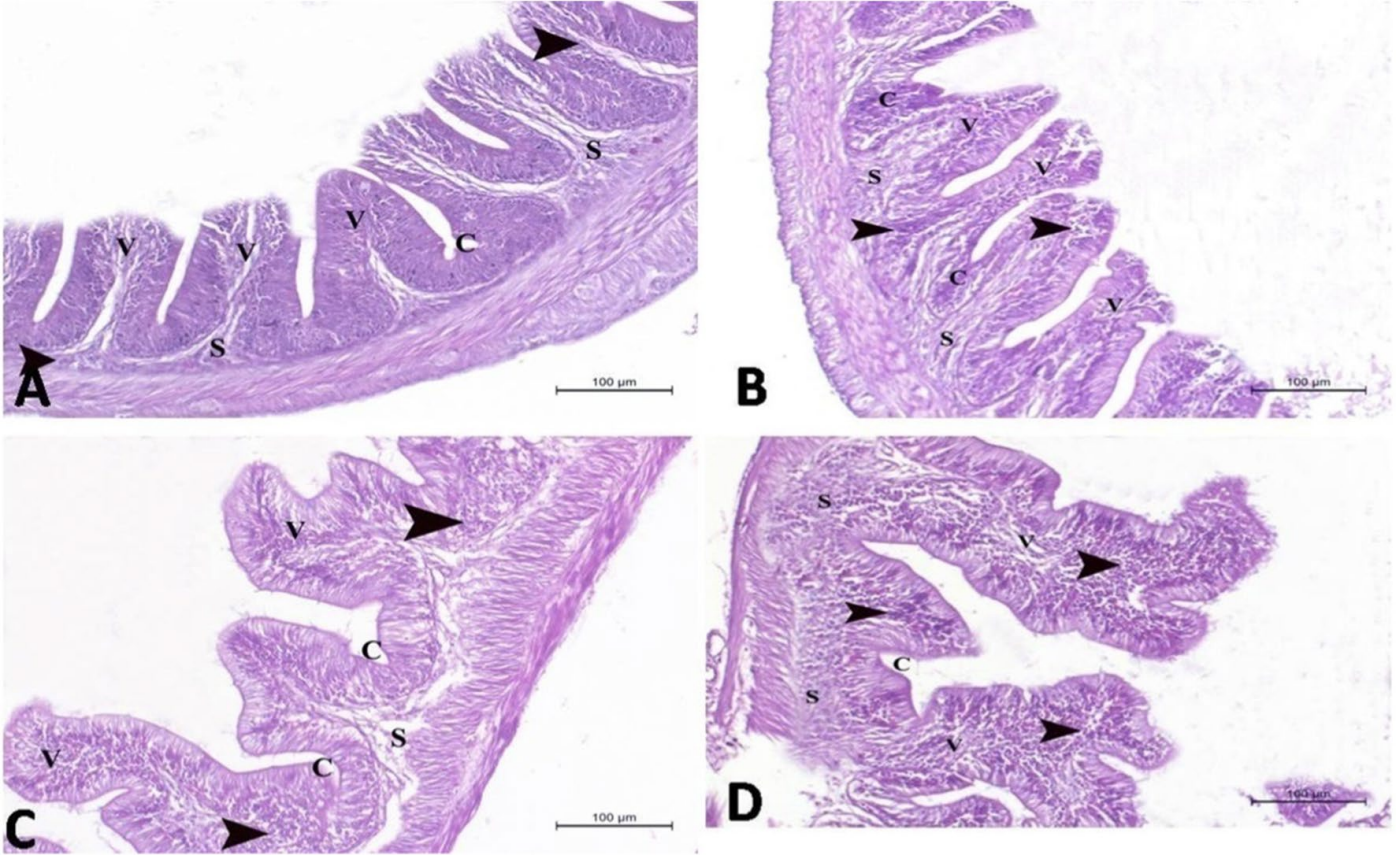
A photomicrograph of the distal segment of intestine in Nile tilapia (H&E stain × 200) in **A** control group, **B** 2.5% Spirulina group, **C** 5.0% Spirulina group, and **D** 10.0% Spirulina group showing normal histological structure of the distal part of intestine with normal intestinal villi (V) of the intestinal mucosa which lined with columnar epithelium and numerous goblet cells. The intestinal crypts (C) appeared in the submucosa (S) which contain massive aggregations of lymphocytes (arrow head). Note: the intestinal villi were increased in the length and width in **B**, **C**, and **D** groups, respectively, when compared to the control group. On the same way, the lymphocytes aggregations were increased in density in **B**, **C**, and **D** groups, respectively, when compared to the control

**Fig. 3:**
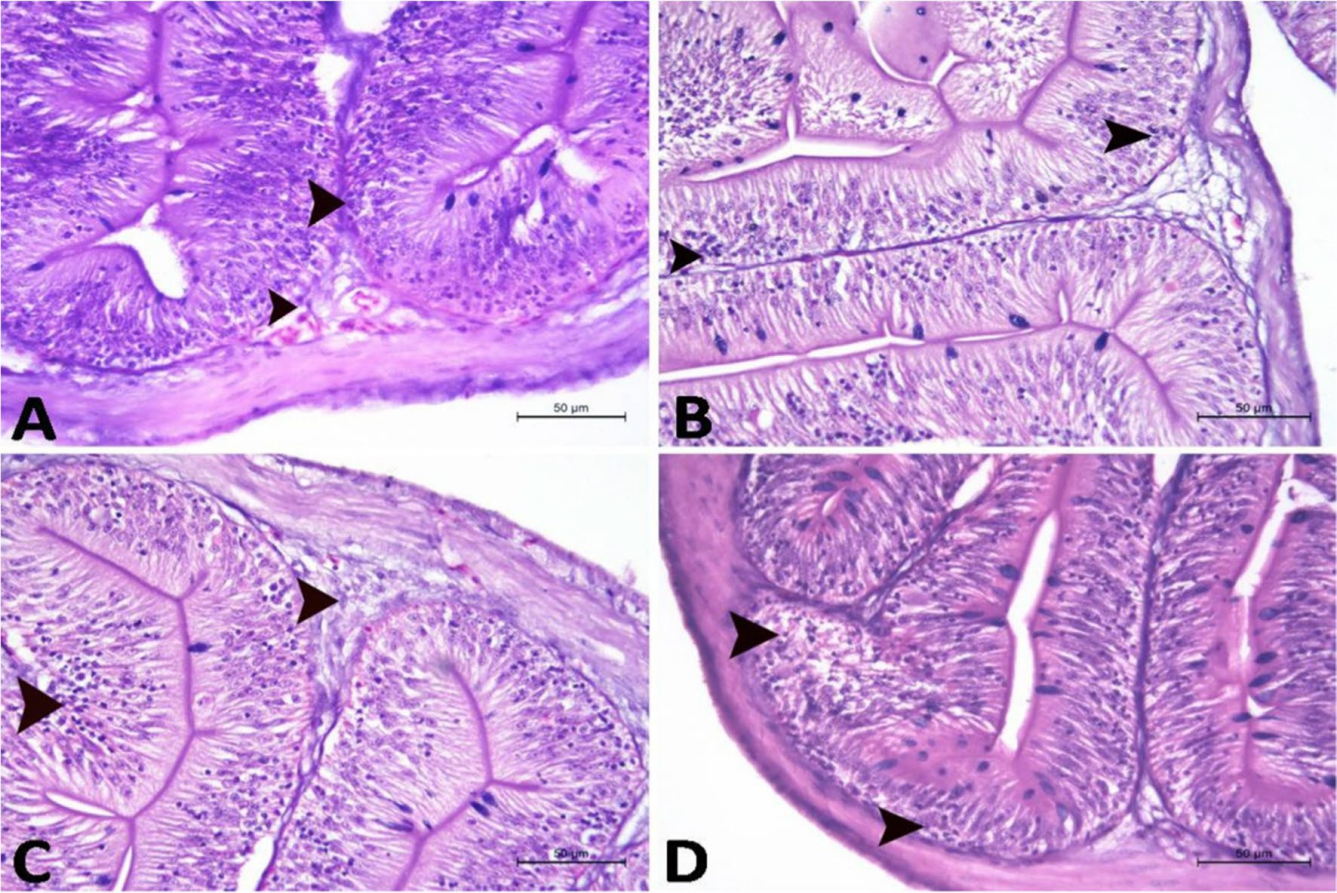
A photomicrograph of the submucosa in proximal segment of intestine in Nile tilapia (H&E stain ×200) in **A** control group, **B** 2.5% Spirulina group, **C** 5.0% Spirulina group, and **D** 10.0% Spirulina group showing lymphocytes (arrow head). The lymphocytes aggregations were increased in density in **B**, **C**, and **D** groups, respectively, when compared to the control. Note: the concentrations of lymphocytes were fewer than that of distal intestinal segment

**Fig. 4:**
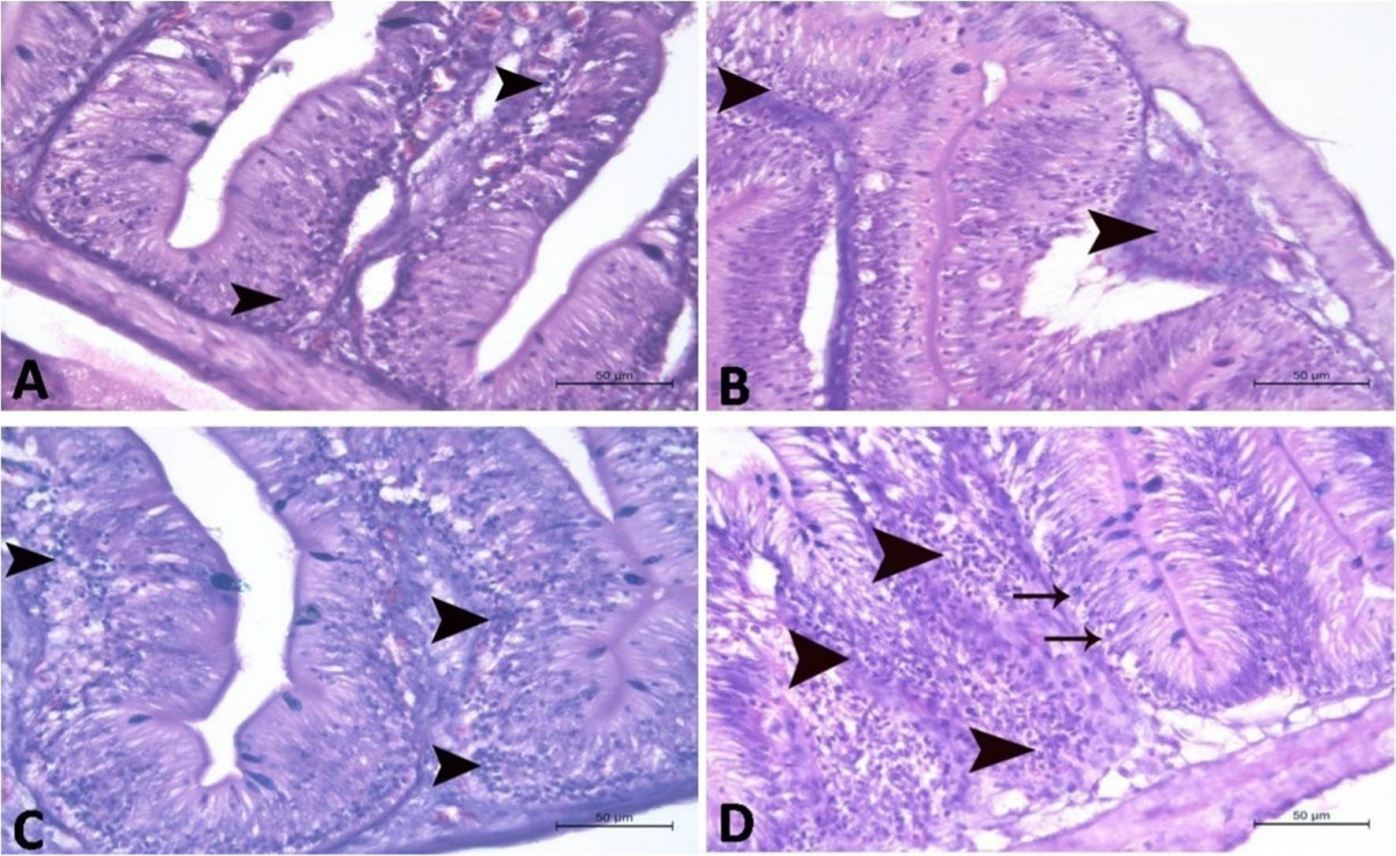
A photomicrograph of the submucosa in distal segment of intestine in Nile tilapia (H&E stain ×200) in **A** control group, **B** 2.5% Spirulina group, **C** 5.0% Spirulina group, and **D** 10.0% Spirulina group showing massive aggregations of lymphocytes (arrow head). The lymphocytes aggregations were increased in density in **B**, **C**, and **D** groups, respectively, when compared to the control. Note: appearance of trans- epithelial lymphocytes in the D group (arrow head)

It was noticed that supplementation of the diet with *S. platensis* increased significantly (*P* < 0.05) the height and width of the intestinal villi, lymphocytes, and goblet cells in the proximal and distal segments of the intestine (Table 7; Figs. 1, 2, 3, and 4). However, the Spirulina algae decreased (*P* < 0.05) the crypt depth of the intestine when compared to the control group. Therefore, the ratio between the villus height and crypt depth was higher (*P* < 0.05) in Spirulina-supplemented groups in comparison to the control one. It was observed that the obtained intestinal findings were influenced by the dietary level of Spirulina, which increased with increasing its level in the diets.

**Table 7:** Intestinal histomorphometry and immunity of Nile tilapia fed diets containing graded levels of *Spirulina platensis*

Parameters	Group				*P* value
	Spirulina levels in the diet (%)				
	0	2.5	5.0	10.0	
Proximal intestine					
Villus height (μm)	800.94 ± 29.87 ^d^	982.75 ± 18.31 ^c^	1072.6 ± 16.84^b^	1414.4 ± 46.75^a^	< 0.001
Villus width (μm)	179.37 ± 6.71 ^d^	232.65 ± 5.04 ^c^	302.91 ± 4.87 ^b^	358.34 ± 7.55 ^a^	< 0.001
Crypt depth (μm)	325.36 ± 21.91 ^a^	289.99 ± 7.40 ^b^	209.10 ± 11.58 ^c^	162.93 ± 9.25 ^d^	< 0.001
Villus height/crypt depth ratio	2.461 ± 0.04 ^d^	3.400 ± 0.09 ^c^	5.129 ± 0.11 ^b^	8.699 ± 0.32 ^a^	0.028
Lymphocytes (cell/microscopic field)	19.800 ± 1.15 ^d^	30.600 ± 1.63 ^c^	43.400 ± 1.56 ^b^	56.200 ± 2.05 ^a^	< 0.001
Goblet cells (cell/microscopic field)	2.000 ± 0.44 ^d^	4.800 ± 0.37 ^c^	8.600 ± 0.50 ^b^	11.400 ± 0.50 ^a^	< 0.001
Distal intestine					
Villus height (μm)	414.19 ±12.10 ^d^	581.98 ± 42.61 ^c^	660.38 ± 10.02^b^	805.3 ± 45.98^a^	< 0.001
Villus width (μm)	237.71 ± 10.43 ^c^	239.08 ± 25.70 ^c^	276.91 ±13.91 ^b^	343.03 ± 13.60 ^a^	< 0.001
Crypt depth (μm)	346.02 ± 9.47 ^a^	275.51 ± 7.70 ^b^	223.43 ± 8.44 ^c^	172.25 ± 3.62 ^d^	< 0.001
Villus height/crypt depth ratio	1.197± 0.00 ^d^	2.112 ± 0.01 ^c^	2.955 ± 0.01 ^b^	4.675 ± 0.02 ^a^	0.021
Lymphocytes (cell/microscopic field)	35.6 ± 1.50 ^d^	45.00 ± 1.304 ^c^	65.200 ±1.855 ^b^	82.800 ± 1.85 ^a^	< 0.001
Goblet cells (cell/microscopic field)	6.200 ± 0.37 ^d^	8.600 ± 0.50 ^c^	11.800 ± 0.37^b^	14.20 ± 0.37 ^a^	< 0.001

^a,b^Means within the same row with different superscripts are significantly different (*P* > 0.05)

## Discussion

Spirulina is considered to be a useful ingredient in the control and prevention of diseases. Moreover, the strength of Spirulina appears to lie in its capability to enhance growth performance, survival rate, and non-specific immune function against fish pathogens, besides its chemo-protective efficiency.

### Growth performance

In the present study, the body weight, weight gain, and specific growth rate of fish fed Spirulina were significantly higher than that fed the control diet. The body weight and weight gain were found to be increased with increasing the inclusion level of Spirulina in the diets. The positive effect of Spirulina on the growth could be due to its high content of protein, amino acids, vitamins, and minerals (Khalila et al., 2018). In addition, Spirulina could increase the fish appetite, resulting in an increased feed intake, and consequently improved the growth (Abdel–Tawabe et al., 2008). Moreover, Spirulina addition in fish diets increased the intestinal villi length and width leading to improved absorption of nutrients (Al-Deriny et al., 2020). Thus, Spirulina, even at low inclusion rates, could provide the nutritional requirements for Nile tilapia without causing adverse effect on its growth and feed efficiency (Olvera-Novoa et al., 1998; Zhang et al., 2020). The same results were found by previous studies (El-Sheekh et al. 2014; Abo El-Ward et al., 2016; Velasquez et al., 2016). In contrast, there was no growth enhancement under low Spirulina inclusion levels (5–20%) in the hybrid red tilapia (*Oreochromis niloticus* × *O. mossambicus*; Ungsethaphand et al., 2010). Also, other studies reported no beneficial effects of dietary Spirulina on growth rate of common carp (*Cyprinus carpio*; Nandeesha et al., 1998) and Nile tilapia (*O. niloticus*; Lu and Takeuchi, 2002; Wan et al., 2021).

The length of fish behaved similarly to the body weight, and it was the highest in 10.0% Spirulina-supplemented group, followed by 5.0%, and then 2.50% treatment. A positive relationship between the fish weight and the body length is indicated, as detected by Saleh et al. (2021). There was a significant increase in the condition factor of tilapia fed Spirulina when compared to the control treatment. Ibrahem et al. (2013) noticed that the condition factor of tilapia was significantly higher in the *S. platensis* groups (5–20%) than in the control one. However, Sirakov et al. (2012) found that the condition factor of tilapia fed 10.0% *S. platensis*-supplemented diets was non-significantly higher than that of the control group.

The feed intake of Spirulina-supplemented groups was found to be slightly higher than that of the control. Similar results have been reported by Al-Zayat (2019) who showed a higher feed intake rate of tilapia fed 2.5–7.5% *S. platensis* when compared to the control. Also, Khalila et al. (2018) reported an increase in feed intake of tilapia fed 0.5% Spirulina. The higher feed intake in Spirulina-supplemented diets might be due to the high protein (50 to 70%) content of Spirulina with a good amino acid profile, besides several nutrients content especially vitamins, minerals, pigments, and carbohydrates (Zhang et al., 2020), which could increase the palatability of the diets. In addition, the better feed intake in fish fed on Spirulina-contained diets may be due to the increased fish appetite and therefore improved the growth (Abdel –Tawabe et al., 2008). However, Pókniak (2007) found that 5.0% of Spirulina meal can be incorporated in the feed for rainbow trout fry without a significant effect on the feed intake level. The FCR was found to be lower in fish fed diets supplemented with Spirulina than the control. The better FCR could be due to that addition of Spirulina in fish diets improved the feed utilization (Hossain et al., 2017). This finding is compatible with Belal and El-Hais (2012) who showed that Nile tilapia (*O. niloticus*) fed diets supplemented with 1.0% Spirulina had significantly improved FCR than those fed the control diet. Khalila et al. (2018) reported a reduction in FCR of tilapia fed 0.5% Spirulina than others. In contrast, the FCR of the experimental fish was not affected by the presence of Spirulina meal (10.0%) in the diet (Sirakov et al., 2012). Also, Ahmadzadenia et al. (2011) showed no significant differences in the FCR between the different experimental variants when replacing soybean meal with Spirulina at rates of 20–80%. It was found that supplementation of Spirulina increased the PER of tilapia fish, indicating improved feed utilization by these algae (Hossain et al., 2017). This result is supported by the findings of previous research (Tan et al. 2017; Khalila et al. 2018; Al-Zayat, 2019).

### Hematological, biochemical, and immunological blood analyses

The hemoglobin and red blood cells count were significantly higher in the Spirulina-supplemented groups than the control group. The same results were reported by El Gammal et al. (2010) and El-Sheekh et al. (2014). Increased RBCs count may be due to Spirulina has 14% phycocyanin which stimulates the erythropoietin hormone production for hematopoiesis (Abdalla et al., 2014). In contrast, RBCs of carp fed 3.0–5.0 g/kg Spirulina were not affected by the dietary treatments (Abdulrahman et al., 2019). The PCV concentration was also increased by feeding of Spirulina. This result is supported by that of El Gammal et al. (2010) and Hegazi et al. (2014) who revealed that the PCV of tilapia fed 10.0–15.0% Spirulina was significantly higher than the control group. Feeding of Spirulina increased also the WBCs count. The same results were found by Hegazi et al. (2014) and Sayed and Fawzy (2014). This increase in WBCs count could be due to the presence of C-phycocyanin in the Spirulina algae, which can help in building the immune capacity (Vonshak, 1997). These results indicate an improvement of fish health when fed Spirulina-supplemented diets, because Spirulina contains carotenoids which increase the ability to fight off infections through the reduction of stress levels (Wu et al., 2016). In addition, the major functions of WBCs are to fight infection and protect the body against foreign organisms (Sayed and Fawzy, 2014).

The serum total protein, globulin levels, and A/G ratio were not affected by the dietary treatments. However, feeding of 10.0% Spirulina significantly increased the albumin serum level, which was also observed by Abdel-Tawwab and Ahmad (2009). The raise in serum albumin level could be due to Spirulina has several active compounds, such as carotenoids, polysaccharides, vitamins, minerals, and linoleic acid, which act as immunostimulants to enhance the immune system (Abdel-Daim et al., 2020). The obtained findings are supported by the results of Al-Deriny et al. (2020) who noticed that 1.0 g/kg Spirulina in tilapia diets did not affect the serum level of total protein, globulin, and A/G ratio. On contrary, 2.0–6.0% Spirulina in gourami fish diets significantly increased the serum levels of total protein, globulin, and A/G ratio (Simanjuntak et al., 2018). There was no significant difference in the glucose concentration among the experimental groups. The same finding was observed by El Gammal et al. (2010) and Al-Deriny et al. (2020). The serum cholesterol and triglycerides levels in Spirulina-supplemented treatments were similar to that of the control group. Al-Deriny et al. (2020) found that 1.0 g/kg Spirulina in tilapia diets did not affect the serum level of total cholesterol and triglycerides. However, 10.0% Spirulina in tilapia diets significantly increased the serum levels of cholesterol and triglycerides (Abdel-Tawwab and Ahmad, 2009). There was no significant difference in serum GOT and GPT levels among the treatments, indicating the liver functions were not affected by the dietary treatments. The same findings were reported by Velasquez et al. (2016) and Al-Deriny et al. (2020). On contrary, Hegazi et al. (2014) observed that addition of 10.0–15.0% *S. platensis* in tilapia diets significantly decreased the serum levels of these enzymes. However, Abo El-Ward et al. (2016) found that 5.0–20.0% Spirulina increased the serum levels of AST and ALT in tilapia. Supplementation of Spirulina significantly decreased the serum levels of urea and creatinine. This refers to the protective effects of Spirulina to keep the fish kidney function in healthy status (Mokhbatly et al., 2020). This is compatible with El Gammal et al. (2010) who recorded that the addition of 5.0–15.0% Spirulina in tilapia diets significantly decreased the serum urea and creatinine levels.

Regarding to the immunological blood parameters, this study revealed that supplementation of Spirulina increased the blood levels of lymphocytes and eosinophils. Abdalla et al. (2014) noticed that lymphocytes and eosinophils of tilapia fed 0.5–1.0% Spirulina numerically increased than that of tilapia fed the control diet. Moreover, 5.0 g/kg Spirulina in carp diet numerically increased the blood levels of these cells (Abdulrahman et al., 2019). The high levels of WBCs, lymphocytes, and eosinophils in Spirulina groups may be due to leucocytes that are centrally involved in the phagocytic activity and as immune responses to parasitic, bacterial, viral, and similar challenges (Houstan, 1990). Thus, the increase in the total leucocytic count, eosinophils, and lymphocytes in fish fed Spirulina-supplemented diets can be attributed to the non-specific immune response, and the increase in lymphocytes may be a specific pathogen-induced immune response (Abdalla et al., 2014). In the present study, supplementation of *S. platensis* did not affect the number of neutrophils and monocytes in fish blood. These results are supported with that of previous studies (El Gammal et al., 2010; Khalil et al., 2017; Abdulrahman et al., 2019).

The IgM level and lysozyme activity in blood of tilapia fed Spirulina were higher than that of fish fed the control diet. This is compatible with Amer (2016) who noticed that 0.5% Spirulina in tilapia diets increased the lysozyme activity and IgM. Also, the IgM of tilapia fed 1.0 g/kg Spirulina was increased when compared to the control group (Al-Deriny et al., 2020). The increased IgM is a direct result of enhanced immunity in tilapia body, probably through increasing the local intestinal immunity (Kiron, 2012). Promya and Chitmanat (2011) reported that Spirulina enhanced the responses of fish lysozyme activity. The increase in the lysozyme activity assay could be due to the presence of C-phycocyanin in the Spirulina algae, which can help in building the immune capacity. In the present study, the phagocytic activity of tilapia fed 10.0% Spirulina was significantly higher than that of fish fed the control diet. Moreover, 2.50 and 5.0% Spirulina increased numerically the phagocytic activity of tilapia. Ragap et al. (2012) found that 10.0 mg/kg Spirulina in tilapia diets significantly elevated the phagocytic activity. In addition, 0.5% Spirulina in African catfish diet significantly increased the phagocytic activity (Mokhbatly et al., 2020). Phagocytosis is regarded as the first cellular line of defense in vertebrates and invertebrates (Chi et al., 2014). Moreover, dietary *S. platensis* has been reported to enhance the phagocytic activity in carp (Watanuki et al., 2006).

### Intestinal histomorphometry and immunity

Supplementation of Spirulina to the diets increased the intestinal villi height and width as well as lymphocytes and goblet cells counts, but reduced the crypt depth of the intestine. Moreover, the Spirulina-supplemented groups showed a higher villi height to crypt depth ratio. This result is supported with the finding of Al-Deriny et al. (2020) who reported that fish fed 1.0 g/kg *S. platensis* exhibited beneficial effects on the intestinal villi length and width as well as the number of goblet cells. The results of Rombout et al. (2011) confirmed the potential role of *S. platensis* in enhancing the absorption capacity of dietary nutrients by the intestinal membranes. Furthermore, the increased number of goblet cells is associated with its role in protecting the intestinal barriers from the pathogenic microorganisms through the secretion of glycoproteins and antibacterial substances (Knoop and Newberry, 2018). Moreover, Shalata et al. (2021) found that supplementation of 1.0 g/kg Spirulina to tilapia diets increased the number of the intra-epithelial lymphocytes. Also, Sheikhzadeh et al. (2019) recorded an increase in the intestinal lymphocytes in rainbow trout fed 2.50–5.0% *S. platensis*.

## Conclusion

The results indicate that using of *S. platensis* in fish diets can enhance the growth performance of tilapia, and it can reduce the dietary fish meal content without affecting the performance. Moreover, Spirulina increased the height and width of intestinal villi, resulting in high absorption of dietary nutrients. Supplementation of Spirulina to fish diets stimulated the immunity of fish through increasing the level of immunological blood indicators (IgM, lysozyme, phagocytic activity, lymphocytes, and eosinophils) as well as the local intestinal immunity (lymphocytes and goblet cells). So, it can be recommended to use *S. platensis* in fish diets not only to improve the growth performance but also to enhance the immune status.

## Data Availability

The data of this study are available from the corresponding author upon reasonable request.
